# Sustainable Pavement Materials: Design, Application, and Performance Evaluation

**DOI:** 10.3390/ma18122783

**Published:** 2025-06-13

**Authors:** Jiaqi Chen

**Affiliations:** Department of Civil Engineering, Central South University, Changsha 410075, China; chenjiaqi@csu.edu.cn

The study of pavement sustainability integrates environmental, economic, and social considerations across the pavement life cycle, with material selection profoundly influencing durability, resource efficiency, safety and maintenance strategies. As global demand for sustainable infrastructure grows, recent research has prioritized innovative road materials and design methodologies to enhance pavement sustainability. Despite significant progress, challenges such as optimizing material performance, reducing environmental footprint, and ensuring cost-effectiveness persist, necessitating further exploration.

This Special Issue—Sustainable Pavement Materials: Design, Application, and Performance Evaluation—in *Materials* (2024–2025) presents eleven research articles that advance sustainable pavement technologies, addressing durability, environmental impact, and resource efficiency in road engineering. In their work, Ma et al. [1] employed multi-scale finite element modeling to reveal that coupled salt–thermal–mechanical effects reduced the cohesive strength of asphalt pavements by 23.5~63.8% and the adhesive strength by 25~71.6%, emphasizing the need for durable designs in harsh environments. Li et al. [2] found that aging and salt–alkali coupling effects decreased the fatigue life and self-healing properties of graphene oxide-modified asphalt, critical for saline–alkaline and high-altitude regions. Meanwhile, Zhou et al. [3] demonstrated that industrial animal oil regenerated aged SBS-modified asphalt, reducing sulfoxide index by up to 38.88% and aromaticity index by up to 63.77%, enhancing recyclability.

In cementitious materials, Li et al. [4] developed sulfur-modified alite calcium sulfoaluminate cement, which achieved 30.45% higher compressive strength at 1 day compared to Portland cement, providing an environmentally friendly alternative. Tang et al. [5] showed that calcined coal-series kaolinite and limestone, replacing 45% of Portland cement, produced a compressive strength of 47.2 MPa at 28 days, improving pore structure and lowering CO_2_ emissions. In addition, Zhang et al. [6] enhanced calcined coal gangue-blended cement with Stöber nano-SiO_2_ particles, increasing compressive strength and refining microstructure. Li et al. [7] improved the compressive strength of cement-stabilized iron tailing soil by using ionic soil stabilizers to increase compactness, yielding an increase of 35.3% after 7 days of testing.

Rudziewicz et al. [8] optimized foam polypropylene fiber-reinforced concrete for 3D printing, achieving a compressive strength of 28.76 MPa with cement of reduced CO_2_ emissions. Gierasimiuk et al. [9] conducted a comparative study on texture measurement techniques for exposed aggregate concrete pavements, providing a reliable assessment of skid resistance. Luo et al. [10] optimized cement content detection in controlled low-strength soils, achieving errors below 2.4% by analyzing water content and hydration time effects. Hou et al. [11] developed a cement-emulsified asphalt composite for negative-temperature spraying, which exhibited superior frost resistance, making it suitable for plateau environments.

Collectively, these studies advance sustainable pavement solutions through their exploration of innovative materials, recycling options, and robust performance evaluation, laying the foundation for durable, eco-friendly road infrastructure. To further their adoption, future research should focus on long-term field performance, cost-effectiveness, and standardized testing.
